# Primary Borderline Mucinous Testicular Tumor: A Case Report and Literature Review

**DOI:** 10.3389/fonc.2020.619774

**Published:** 2021-03-09

**Authors:** Changjuan Hao, Chunsong Kang, Xiaoyan Kang, Zhuanzhuan Yu, Tingting Li, Jiping Xue

**Affiliations:** ^1^ Department of Ultrasound, Shanxi Bethune Hospital affiliated to Shanxi Medical University, Taiyuan, China; ^2^ Department of Pathology, Shanxi Bethune Hospital affiliated to Shanxi Medical University, Taiyuan, China

**Keywords:** testis, mucinous tumor, clinical characteristic, imaging characteristic, differential diagnosis

## Abstract

Primary mucinous tumors of the testis and paratestis are very rare, with only 29 reported cases detected in a PubMed search. The histopathological characteristics of primary testicular mucinous tumors are similar to their ovarian counterparts, and the diagnosis and naming criteria refer to the criteria for female ovarian mucinous tumors. However, the clinical and imaging features of primary testicular mucinous tumors are poorly understood, and they are thus frequently undiagnosed or misdiagnosed. We present the case of a patient with a primary testicular mucinous tumor. A 52-year-old man presented with a 1-year history of painless enlargement of the left scrotum. Ultrasound examination revealed a cystic mass in the left testis, with viscous fluid areas and calcified spots, irregular solid bulges on the cyst wall, and a small blood supply. Serum alpha-fetoprotein, β-human chorionic gonadotropin, lactate dehydrogenase, renal function, inflammatory markers, and routine urine and blood examinations were all normal. The patient underwent radical resection of the left testis. Postoperative pathology showed a multilocular cystic mass, with the inner wall of the sac lined with mucous columnar epithelial cells, some with mild nuclear atypia, and no interstitial infiltration. The pathological diagnosis was testicular mucinous tumor. Postoperative abdominal and pelvic computed tomography, colonoscopy, and gastroscopy showed no suspicious lesions. The final diagnosis was primary testicular borderline mucinous tumor. The patient underwent postoperative follow-up examinations once a year for 4 years. Serum tumor markers, scrotal ultrasound, abdominal and pelvic computed tomography scans, and colonoscopy and gastroscopy revealed no evidence of metastases or other primary adenocarcinoma. This case highlights the clinical and imaging characteristics of primary testicular mucinous tumors, which might aid their differential diagnosis.

## Introduction

Primary tumors of the testis and paratesticular tissue include various pathological types. Germ cell tumors are the most frequent, while ovarian epithelial tumors are rare, and mucinous tumors are extremely rare, with only 29 reported cases detected in a PubMed search (1–3). The histopathological characteristics of testicular mucinous tumors are similar to those of ovarian mucinous tumors, and their diagnosis and naming standards thus refer to the diagnostic criteria for female ovarian mucinous tumors (1, 2). However, the rarity of primary mucinous tumors of the testis means that clinicians have insufficient knowledge of their clinical and imaging features. Their preoperative diagnosis is thus difficult, and such tumors are therefore often undiagnosed or misdiagnosed. Previous studies have focused on their tissue origin and their similarities and differences with ovarian mucinous tumors, but reports of their clinical and imaging characteristics are lacking (4–25). We report the case of a patient with a primary testicular mucinous tumor and review the related literature, to raise clinicians’ awareness of the clinical characteristics, imaging features, and key diagnostic points of this rare type of tumor.

## Case Description

A previously healthy 52-year-old man presented with a 1-year history of painless left scrotum enlargement and mild discomfort, but no lower urinary tract symptoms. He was admitted 1 month after his symptoms worsened, with subjective fever, but still without lower urinary tract symptoms. Before admission to hospital, he had been treated at a local community hospital and had received oral empiric antibiotic levofloxacin for 2 weeks, with no significant improvement in his symptoms. The patient had no history of cryptorchidism, scrotal trauma, scrotal inflammation, or urinary tuberculosis, and no history of neoplastic disease or family history of genetic diseases.

**Figure 1:**
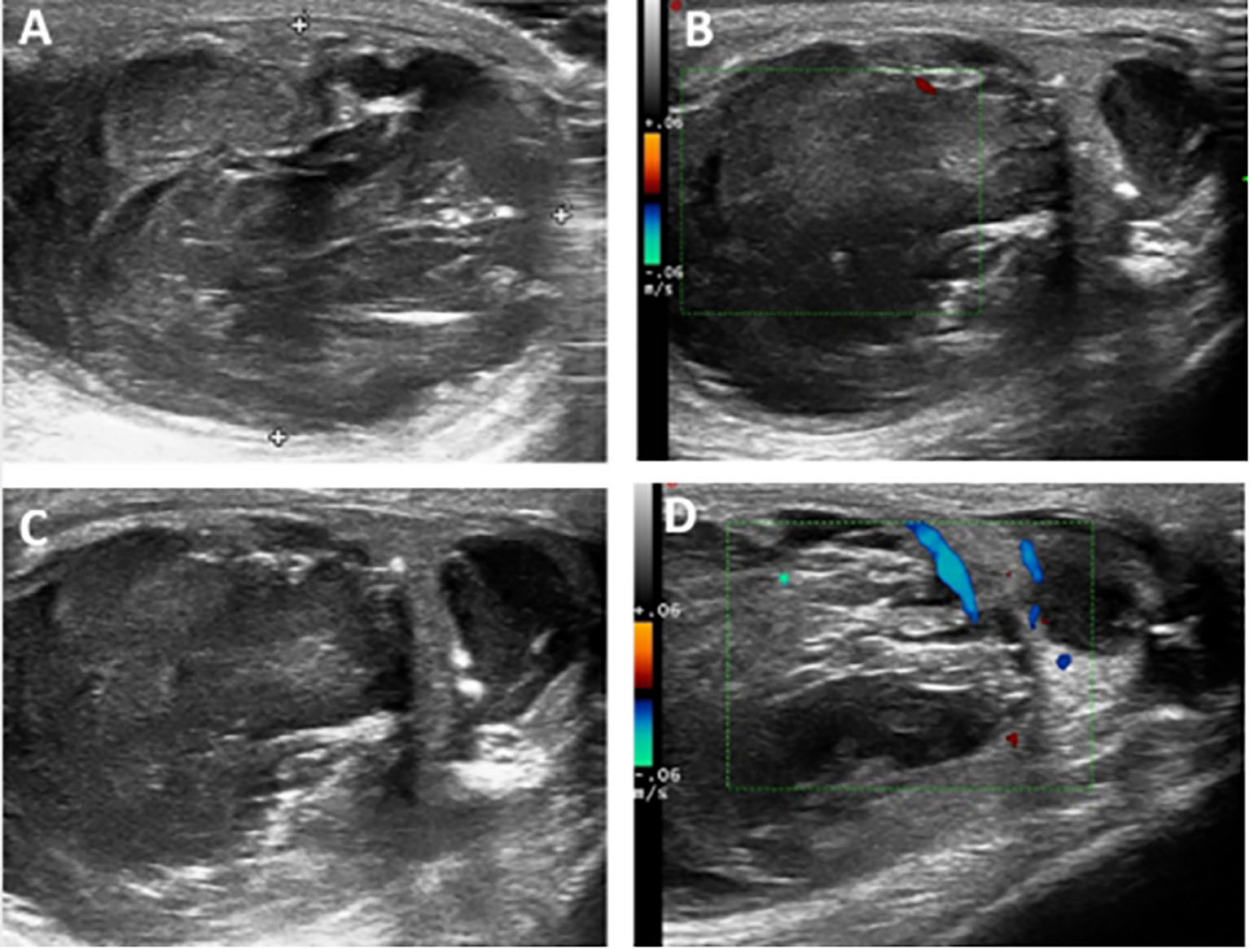
**(A)** Ultrasound examination of the scrotum revealed a mainly cystic, heterogeneous echogenic mass occupying most of the left testicle. The tumor had a clear boundary, regular morphology, and disordered internal echo. **(B, C)** Viscous fluid was seen in the cystic part, and large calcified spots were detected in some areas. **(D)** A few irregular solid protrusions were visible on the cyst wall with a small blood supply.

Physical examination of his testes revealed an enlarged, hard, and non-tender left testis, a clinically normal right testis, and no palpable inguinal lymphadenopathy. Ultrasound examination of the scrotum revealed a mainly cystic, heterogeneous echogenic mass occupying most of the left testicle. The tumor had a clear boundary, regular morphology, and disordered internal echo (**Figure 1A**). Viscous fluid was seen in the cystic part, and large calcified spots were detected in some areas (**Figures 1B, C**). A few irregular solid protrusions were visible on the cyst wall with a small blood supply (**Figure 1D**). There were no obvious enlarged lymph nodes in the groin area on either side. The ultrasound findings suggested a testicular tumor or abscess. However, the testicular tumor marker serum alpha-fetoprotein (AFP) was 2.2 ng/mL (normal reference value 0–20 ng/mL), β-human chorionic gonadotropin (β-HCG) was 0.6 mIU/mL (normal reference value 0.5–2.67 mIU/mL), and lactate dehydrogenase (LDH) was 136.3 IU/L (normal reference value 120–250 IU/L). Renal function, inflammatory markers, and routine urine and blood tests were all normal. We therefore decided to perform exploratory surgery of the left testicular mass.

**Figure 2:**
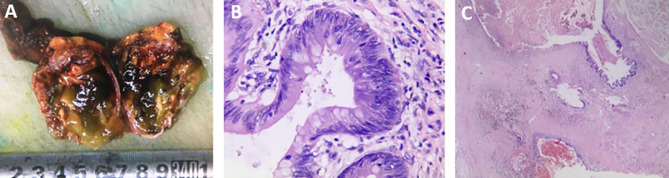
**(A)** Gross pathology showed that the multilocular cystic mass in the left testis occupied almost the entire testicular parenchyma (volume about 4.5×3.5×2.5 cm) with irregular thickening of the cyst wall and filled with gray-yellow jelly. **(B)** Microscopic pathology (hematoxylin and eosin staining ×200) showed that the inner wall of the capsule was lined with a single layer of pseudo-stratified mucus columnar epithelial cells, some showing mild nuclear atypia, but with no interstitial infiltration. **(C)** Microscopic pathology (hematoxylin and eosin staining ×20) also showed fibrous tissue proliferation of the cyst wall and mucus overflow into the cyst wall in some areas.

Surgery revealed a multilocular cystic mass in the left testis. There was no abnormality in the left epididymis or spermatic cord. Intraoperative frozen pathology indicated a testicular mucinous tumor, and radical resection of the left testis was performed. Postoperative gross pathology showed a multilocular cystic mass in the left testis measuring about 4.5×3.5×2.5 cm and occupying almost the entire testicular parenchyma, with irregular thickening of the cyst wall and filled with gray-yellow jelly (**Figure 2A**). Microscopy showed that the inner wall of the capsule was lined with a single layer of pseudo-stratified mucus columnar epithelial cells, some showing mild nuclear atypia, but with no interstitial infiltration. There was also fibrous tissue proliferation of the cyst wall and mucus overflow into the cyst wall in some areas (**Figures 2B, C**). The cyst cavity was filled with mucus, with focal calcification in some areas, but no teratoma component. Based on the pathological characteristics, the diagnosis was primary or secondary testicular mucinous tumor.

On the basis of this unexpected pathological finding, a further diagnostic work-up was carried out, including abdominal and pelvic computed tomography (CT) and upper and lower gastrointestinal tract endoscopy, but no other suspicious primary tumors or metastases were found. The tumor was thus finally diagnosed as a primary borderline mucinous tumor of the testis, according to the 2016 edition of the WHO Classification of Tumors of the Urinary System and Male Reproductive Organs (1).

The patient underwent postoperative follow-up examinations once a year for 4 years after surgery. His serum tumor markers remained at normal levels, and scrotal ultrasound, abdominal and pelvic CT scans, and colonoscopy and gastroscopy revealed no evidence of metastases or any other primary adenocarcinoma. His postoperative recovery was uneventful.

Patient perspective: “I was previously healthy with no known diseases. Before coming to the hospital, I had had a 1-year history of painless left scrotum enlargement and mild discomfort, and had felt the symptoms worsening for more than a month. After coming to the hospital and undergoing left orchiectomy surgery, my left scrotum discomfort disappeared. My postoperative recovery was uneventful, and there were no abnormal findings during annual postoperative follow-up examinations.”

## Discussion

Ovarian surface epithelial tumors are the most common type of ovarian tumors, and similar tumors can also rarely occur in the testis and adjacent tissue (1–3). Similar to their ovarian counterparts, the entire spectrum of ovarian surface epithelial tumors has been reported in the testis and paratestis, including serous, mucinous, endometrioid, clear cell, and Brenner tumors (1, 2). Serous tumors are the most common, while mucinous tumors are extremely rare (1, 2). Twenty-nine cases of primary testicular or paratesticular mucinous tumors have been reported in the literature to date, all showing histopathological characteristics similar to those of ovarian mucinous tumors, with cystic masses lined with mucous columnar epithelial cells (4–25). The diagnosis and naming standards for testicular mucinous tumors in the 2016 WHO Classification of Urinary System and Male Reproductive Organ Tumors thus refers to the criteria for female ovarian mucinous tumors, which are divided into mucinous cystadenoma, borderline mucinous tumor, and mucinous cystadenocarcinoma (**Table 1**) (1).

**Table 1:** Summary of reported primary testicular/paratesticular mucinous tumors.

	Mucinous cystadenoma (n)	Borderline mucinous tumor (n)	Mucinous carcinoma (n)	Total number (n)
Case (n)	11(37.9%)	10(34.5%)	8(27.6%)	29(100%)
Age (yr)				
≥40 years	6(20.7%)	10(34.5%)	8(27.6%)	24(82.8%)
<40 years	5(17.2%)	0(0.0%)	0(0.0%)	5(17.2%)
Number of testes involved				
Unilateral	11(37.9%)	10(34.5%)	8(27.6%)	29(100%)
Bilateral	0(0.0%)	0(0.0%)	0(0.0%)	0(0.0%)
Amount				
Single	11(37.9%)	10(34.5%)	8(27.6%)	29(100%)
Multiple	0(0.0%)	0(0.0%)	0(0.0%)	0(0.0%)
Tumor location				
Testicular	7(24.1%)	8(27.6%)	7(24.1%)	22(75.9%)
Paratesticular	4(13.8%)	2(6.9%)	1(3.4%)	7(24.1%)
Surgery				
Radical orchiectomy	10(34.5%)	10(34.5%)	8(27.6%)	28(96.6%)
Testicular mass resection	1(3.4%)	0(0.0%)	0(0.0%)	1(3.4%)
Prognosis				
No disease recurrence	8(27.6%)	9(31.0%)	4(13.8%)	21(72.4%)
Recurrence or metastasis	0(0.0%)	1(3.4%)	3(10.4%)	4(13.8%)
Unknown	3(10.4%)	0(0.0%)	1(3.4%)	4(13.8%)

**Figure 3:**
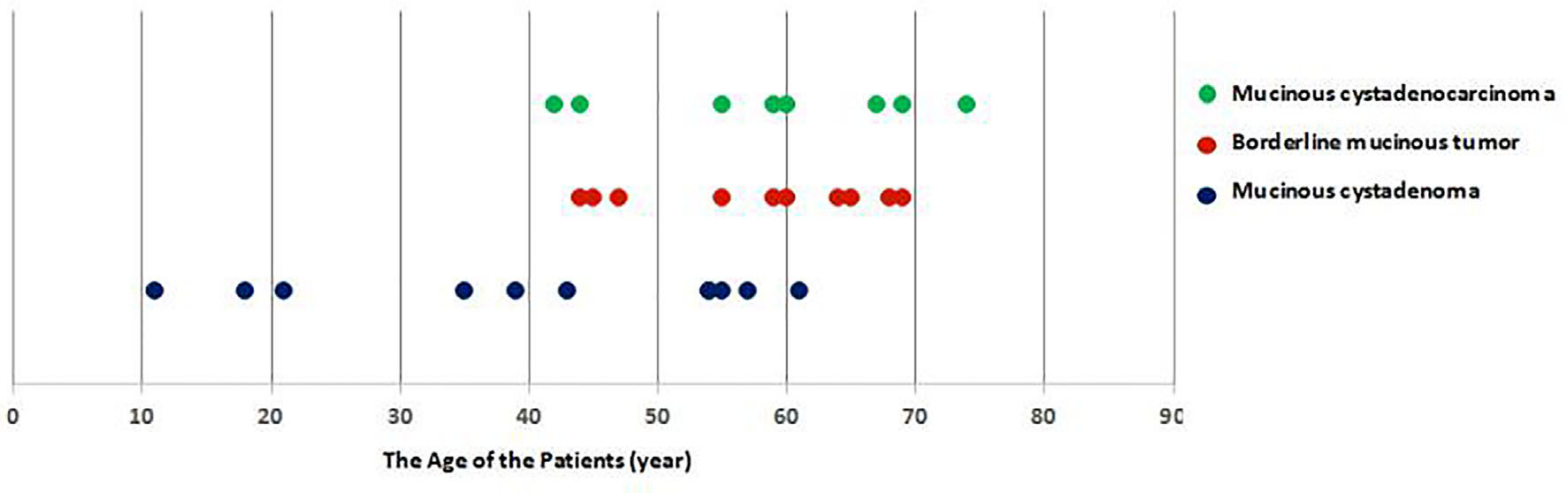
The ages of the 29 previously reported patients ranged from 11–78 years. They included 10 cases of borderline mucinous tumors and eight of mucinous cystadenocarcinoma, all in patients above 40 years old. Mucinous cystadenoma occurred in seven patients older than 40 years old and in five younger than 40 years old. Primary mucinous testicular tumors accounted for 83% (24/29) of patients above 40 years old, compared with 33% (8/24) of patients with mucinous cystadenocarcinoma. mucinous cystadenoma, borderline mucinous tumor, and mucinous cystadenocarcinoma.

Because primary mucinous tumors of the testis are extremely rare, their age prevalence and clinical features are not yet fully understood. The 29 previously reported patients ranged from 11–78 years old (**Figure 3**) (4–25). They included 10 cases of borderline mucinous tumors and eight of mucinous cystadenocarcinoma, all in patients older than 40 years (4, 14–25). Patients with mucinous cystadenoma ranged from 11–78 years, including six over and five under 40 years old (4–13). Primary mucinous testicular tumors accounted for 83% (24/29) of patients over 40 years old, compared with 33% (8/24) of patients with mucinous cystadenocarcinoma. Primary mucinous testicular tumors thus seemed to occur in patients over 40 years old, with nearly a third of patients over 40 years old having malignant tumors. The usual age range for testicular germ cell tumors, as the most common testicular tumor, is 18–40 years, suggesting that patient age may be an indicator of tumor type (1, 2). Similar to other testicular tumors, most patients with primary testicular mucinous tumors lack typical clinical symptoms: 83% (24/29) of reported cases showed painless scrotal swelling, with only 17% (5/29) showing slight tenderness of the scrotum (4–25). The lack of scrotal symptoms in most cases means that the lesions are easily ignored by patients. Maruschke and colleagues reported the case of a 67-year-old patient with a 20-year history of primary testicular mucinous cystadenocarcinoma, who failed to seek medical attention because of the lack of obvious symptoms, but who subsequently developed retroperitoneal lymph node metastasis 2 years after surgery (24). Patients over the age of 40 years with painless scrotal swelling should thus seek medical treatment to rule out primary mucinous tumors of the testis.

Preoperative serum tumor marker detection and imaging examinations can aid the assessment of patients with suspected testicular tumors (3). The main testicular serum tumor markers include AFP, β-HCG, and LDH (1, 3). Some of the reported cases were tested for tumor markers (6, 7, 10–18, 21, 22, 24); however, only one patient with testicular mucocystadenoma had elevated β-HCG (433.37 mIU/mL, normal <3.81), and the levels of all testicular tumor markers were normal in the remaining patients (13). Some patients were also tested for carcinoembryonic antigen (CEA) and carbohydrate antigen 19-9 (CA19-9). Azuma et al. reported a patient with primary mucinous cystadenocarcinoma of the testis whose laboratory findings showed elevated serum CA19-9 (17,200 U/mL, normal <37 U/mL) and CEA (6.5 ng/mL, normal<5 ng/mL), but normal levels of the germ cell tumor markers LDH, β-hCG, and AFP (25). Funada et al. also reported a patient with primary borderline mucinous tumor of the testis who had elevated levels of CEA (12 ng/mL, normal <5 ng/mL) but normal serum LDH, β-hCG, and AFP levels (17). These cases indicate that a lack of elevated testicular tumor markers such as LDH, β-hCG, and AFP does not exclude the existence of a primary testicular mucinous tumor, while increased CA19-9 and CEA levels might provide an early indication of a testicular mucinous tumor, especially mucinous adenocarcinoma or mucinous borderline tumor. The European Association of Urology recommends scrotal ultrasound as the preferred imaging method for testicular tumors, with the ability to clarify the location, size, shape, and cysticity of the tumor, while CT and magnetic resonance imaging of the chest, abdomen, and pelvis can detect metastases (3). Previous cases have indicated that most tumors occur as a predominantly cystic mass in the testis and adjacent tissues, affecting only one side of the testis, and are all single (4–25). Their ultrasound features include clear borders and regular morphology, with calcified plaques visible in some masses (5–7, 9–20, 25). However, ultrasound lacks specificity for distinguishing between benign and malignant lesions, and although the above imaging features differ from those of testicular seminoma and lymphoma, they are difficult to distinguish from metastatic testicular mucinous tumors and teratomas.

Primary testicular mucinous tumors are very rare, lack specific clinical manifestations and serum tumor markers, and have similar imaging characteristics to testicular metastatic mucinous tumors and teratomas, making their differential diagnosis particularly important. Testicular metastatic tumors from the colon, pancreas, or stomach can mimic primary mucinous tumors, resulting in cystic lesions in the testis similar to primary mucinous tumors; however, the prognosis of metastatic mucinous tumors of the testis is extremely poor (4, 26–29), and this possibility should thus be excluded as soon as possible. In patients with no obvious symptoms of primary tumors of the colon, pancreas, or stomach, symptoms such as scrotal swelling caused by metastatic testicular tumors may be the first clinical manifestation. However, the primary tumor in the colon, pancreas, or stomach may be easily ignored, leading to the possibility of misdiagnosis (26–29). Verma et al. reported a case of colon cancer with scrotal swelling as the first clinical manifestation, and Meacham et al., Polychronidis et al., and Tiong et al. have all reported similar cases (26–29). Vermaet et al. thus suggested that patients with a testicular mass should undergo CT scans of the abdomen and pelvis, as well as gastrointestinal endoscopy, to rule out the possibility of testicular metastasis, regardless of whether the patient has a history of malignant tumors (26). Colonoscopy and gastroscopy are not part of the routine diagnostic evaluation or follow-up for testicular malignancies recommended by the European Association of Urology Guidelines for Testicular Tumors (3). However, these guidelines are not inclusive of metastatic deposits from gastrointestinal primaries (3). Given the concern over potential gastrointestinal primary tumors, gastrointestinal endoscopy was deemed a reasonable approach, as the gold standard for detecting gastrointestinal tumors and the ability to provide a tissue diagnosis (14). Vermaet et al. therefore suggested that it was essential to exclude testicular metastatic mucinous gland tumors derived from the digestive tract using gastrointestinal tract endoscopy, to ensure the correct diagnosis of primary testicular mucinous tumors.26 Notably however, gastrointestinal endoscopy is only important in the case of mucinous testicular tumors, and is not appropriate for all testicular masses.

In addition, it is also necessary to rule out germ cell tumors, such as teratomas with prominent mucinous components.The clinical manifestations and imaging characteristics of the two are similar, but the age distributions of primary mucinous tumors and teratomas differ: primary mucinous tumors are more common in patients over 40 years old (median 55 years old), while teratomas rarely occur in patients over 40 years old (median 23–29 years old) (1, 2, 30–32). Pathological teratomas almost always include teratoma components other than mucinous epithelial components under the microscope, and about 90% of teratomas in adults contain germ cell tumors *in situ* in the seminiferous tubules (1, 2). The histopathological characteristics of primary mucinous tumors and teratomas are thus obviously different, allowing a diagnosis to be confirmed.

Reports of treatment experiences for patients with primary testicular mucinous tumors are limited, and there are currently no clear treatment guidelines. Radical orchiectomy is the most commonly used treatment (4–25). Among the currently reported cases, only one 18-year-old patient with a paratesticular mucocystadenoma underwent testicular mass resection, with no tumor recurrence or metastasis after a 1.5-year follow-up (7), and the other patients chose to undergo radical orchiectomy (4–6, 8–25). All the reported cases of patients under 40 years old involved mucinous adenomas, and we therefore suggest that testicular mass resection may be considered for patients younger than 40 years old with fertility requirements. However, their treatment results still need to be followed up. Nevertheless, reports of primary mucinous tumors of the testis are limited, and its prognosis and long-term management are thus largely uncertain and non-standardized, respectively. Of the 29 reported patients, four were lost to follow-up (4–6, 21). Follow-up of the previous 25 cases of primary mucinous tumors of the testis or paratestis (8 mucinous cystadenoma, 10 borderline mucinous tumors, 7 mucinous cystadenocarcinoma) ranged from 8 months to 14 years, with only four cases of recurrence or metastasis (3 mucinous cystadenocarcinoma and 1 borderline mucinous tumor) (4, 24, 25). One patient with mucinous cystadenocarcinoma died 2 months after diagnosis with frankly invasive carcinoma and peritoneal tumor deposits with no other potential primary tumor site identified (4). Another patient with mucinous cystadenocarcinoma died 10 months later following metastasis to the liver, lungs, and retroperitoneal lymph nodes (25). One patient with mucinous cystadenocarcinoma developed retroperitoneal lymph node metastasis 2 years after surgery, discontinued treatment, and was eventually lost to follow-up (24). One patient with a borderline mucinous tumor died of tumor metastasis 12 years after surgery (4). Recurrence and metastasis of mucinous cystadenoma has not been reported (4–13), and based on the small number of reported cases, such tumors within the scrotum appear to share the excellent prognosis of their ovarian counterparts (4, 14). However, because primary mucinous tumors of the testis are extremely rare, the long-term postoperative survival rates of men with analogous tumors are still uncertain. Compared with primary mucinous tumors, metastatic mucinous tumors of the digestive system are more common in the testes (2, 14, 22, 26–29). Pratap et al. thus suggested that scrotal ultrasound, abdominal and pelvic CT, gastrointestinal endoscopy, and testicular tumor markers should be re-examined on a regular basis after surgery (14). In the current patient with a mucinous borderline tumor, scrotal ultrasound, abdominal and pelvic CT, gastrointestinal endoscopy, and serum tumor marker detection revealed no signs of recurrence or metastasis at 4 years after surgery.

In conclusion, the analysis of currently reported cases indicated that primary mucinous testicular tumors tend to occur in people over 40 years old, with nearly a third of patients over 40 years old having malignant tumors; however, their clinical manifestations and tumor markers lack specificity, and the predominant imaging characteristics include a predominantly cystic mass in the testis and adjacent tissues. Metastatic testicular mucinous tumors and teratomas are difficult to distinguish based on their imaging characteristics, and abdominal and pelvic CT examination and gastrointestinal endoscopy are needed to exclude metastatic mucinous tumors. The final diagnosis depends on histopathological examination. Radical orchiectomy is the most widespread treatment for primary mucinous tumors of the testis, and scrotal ultrasound, abdominal and pelvic CT, and gastrointestinal endoscopy should be performed and reviewed regularly after surgery.

## Data Availability Statement

The original contributions presented in the study are included in the article/**Supplementary Material**. Further inquiries can be directed to the corresponding author.

## Ethics Statement

The studies involving human participants were reviewed and approved by appropriate institutional of Shanxi Bethune Hospital. The patients/participants provided their written informed consent to participate in this study. Written informed consent was obtained from the individual(s) for the publication of any potentially identifiable images or data included in this article.

## Author Contributions

CH, ZY, and XK wrote the manuscript. CK revised the manuscript. TL and JX reviewed the manuscript. All authors contributed to the article and approved the submitted version.

## Conflict of Interest

The authors declare that the research was conducted in the absence of any commercial or financial relationships that could be construed as a potential conflict of interest.
