# Supplementary material for: Primary Borderline Mucinous Testicular Tumor: A Case Report and Literature Review
Source: Front Oncol. 2021 Mar 9;10:619774. doi: 10.3389/fonc.2020.619774 (PMC7986722; doi:10.3389/fonc.2020.619774)
Supplement: Supplementary file 1 [file DataSheet_1.pdf]

Timeline

A previously well 52-year-old man presented with a 1-year history of painless left scrotum enlargement and mild discomfort, but no lower urinary tract symptoms.

2/2016

He been treated at a local community hospital and received oral empiric antibiotic levofloxacin for 2 weeks,with no significant improvement in his symptoms.

He was admitted 1 month after his symptoms worsened, with subjective fever, but still without lower urinary tract symptoms.

3/2016

Physical examination: an enlarged, hard, and non-tender left testis, a clinically normal right testis, and no palpable lymphadenopathy

He went to the urology department of our hospital for treatment

Ultrasound examination: a cystic mass in the left testis, with viscous fluid areas and calcified spots, irregular solid bulges on the cyst wall, and a small blood supply

Chemical examination: serum alpha-fetoprotein, β-human chorionic gonadotropin, lactate dehydrogenase, renal function, inflammatory markers, and routine urine and blood examinations were all normal.

4/2016

The patient underwent radical resection of the left testis.

Pathological diagnosis was testicular mucinous tumor.

5/2016

CT and Gastroscopy: Postoperative abdominal and pelvic CT, colonoscopy, and gastroscopy showed no suspicious lesions.

The final diagnosis : primary testicular borderline mucinous tumor

6/2020

His postoperative recovery was uneventful. The patient underwent postoperative follow-up examinations once a year for 4 years after surgery. His serum tumor markers remained at normal levels, and scrotal ultrasound, abdominal and pelvic CT scans, and colonoscopy and gastroscopy revealed no evidence of metastases or any other primary adenocarcinoma.
