# Supplementary material for: Primary Borderline Mucinous Testicular Tumor: A Case Report and Literature Review
Source: Front Oncol. 2021 Mar 9;10:619774. doi: 10.3389/fonc.2020.619774 (PMC7986722; doi:10.3389/fonc.2020.619774)
Supplement: Supplementary file 2 [file DataSheet_2.pdf]

CARE case report guidelines

CARE Checklist of information to include when writing a case report

(cc) BY-NC-ND

Topic	Item	Checklist item description	Reported on Lin
Title	1	The diagnosis or intervention of primary focus followed by the words "case report"	page5
Key Words	2	2 to 5 key words that identify diagnoses or interventions in this case report, including "case report"	page5
Abstract (no references)	3a	Introduction: What is unique about this case and what does it add to the scientific literature?	page5
	3b	Main symptoms and/or important clinical findings	page5
	3c	The main diagnoses, therapeutic interventions, and outcomes	page5-6
	3d	Conclusion—What is the main "take-away" lesson(s) from this case?	page6
Introduction	4	One or two paragraphs summarizing why this case is unique (**may include references**)	page6
Patient Information	5a	De-identified patient specific information.	page6
	5b	Primary concerns and symptoms of the patient.	page6
	5c	Medical, family, and psycho-social history including relevant genetic information	page6
	5d	Relevant past interventions with outcomes	page6
Clinical Findings	6	Describe significant physical examination (PE) and important clinical findings.	page6
Timeline	7	Historical and current information from this episode of care organized as a timeline	page6
Diagnostic Assessment	8a	Diagnostic testing (such as PE, laboratory testing, imaging, surveys).	page6
	8b	Diagnostic challenges (such as access to testing, financial, or cultural)	page6
	8c	Diagnosis (including other diagnoses considered)	page6
	8d	Prognosis (such as staging in oncology) where applicable	page6
Therapeutic Intervention	9a	Types of therapeutic intervention (such as pharmacologic, surgical, preventive, self-care)	page6
	9b	Administration of therapeutic intervention (such as dosage, strength, duration)	page6
	9c	Changes in therapeutic intervention (with rationale)	page6
Follow-up and Outcomes	10a	Clinician and patient-assessed outcomes (if available)	page7
	10b	Important follow-up diagnostic and other test results	page7
	10c	Intervention adherence and tolerability (How was this assessed?)	page9
	10d	Adverse and unanticipated events	page9
Discussion	11a	A scientific discussion of the strengths AND limitations associated with this case report	page9
	11b	Discussion of the relevant medical literature **with references**.	page7-9
	11c	The scientific rationale for any conclusions (including assessment of possible causes)	page9
	11d	The primary "take-away" lessons of this case report (without references) in a one paragraph conclusion	page9
Patient Perspective	12	The patient should share their perspective in one to two paragraphs on the treatment(s) they received	page7
Informed Consent	13	Did the patient give informed consent? Please provide if requested	Yes ☑ No ☐
